# Supplementary material for: Causal Relationship between Aging and Anorexia Nervosa: A White-Matter-Microstructure-Mediated Mendelian Randomization Analysis
Source: Biomedicines. 2024 Aug 16;12(8):1874. doi: 10.3390/biomedicines12081874 (PMC11351342; doi:10.3390/biomedicines12081874)
Supplement: Supplementary file 1 [file biomedicines-12-01874-s001.zip › Supplementary Figure/Figure S8/Leaveoneout plot of MR analysis for TL to ubm-b-1999.pdf]

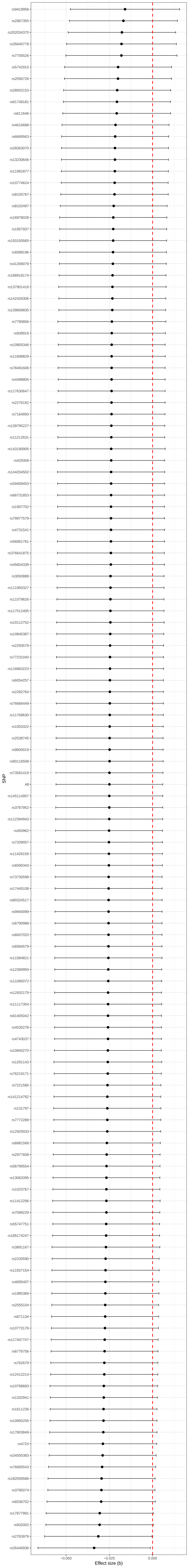

rs9419958
rs2967355
rs202034370
rs35640778
rs7705526
rs5742915
rs2056726
rs28502153
rs61748181
rs611646
rs4616688
rs6669563
rs28363070
rs13230646
rs11991877
rs10774624
rs8105767
rs8102497
rs16978028
rs1957937
rs150150565
rs9398196
rs41269079
rs188918174
rs137901416
rs142426306
rs139669835
rs7790856
rs939916
rs10805346
rs11699829
rs78491606
rs4498805
rs117630647
rs2276182
rs7164950
rs139795227
rs11212631
rs143190905
rs429358
rs144204502
rs59409453
rs66731853
rs1907702
rs79977579
rs4731541
rs56061761
rs376641875
rs45604339
rs3093888
rs111950327
rs11579626
rs117512405
rs10112752
rs10845387
rs2293579
rs77231040
rs116863223
rs6054257
rs2282764
rs76666449
rs11769630
rs1003322
rs2538745
rs9600019
rs80116508
rs73581419
All
rs145114957
rs3767952
rs112394943
rs450962
rs7209057
rs11426156
rs6590343
rs73730598
rs17445108
rs80324517
rs9940099
rs6790988
rs6007020
rs6584579
rs11584821
rs12369950
rs11085072
rs12932179
rs11117354
rs61405042
rs4530278
rs4743037
rs10840270
rs1291143
rs76219171
rs7221585
rs141214782
rs131797
rs7772289
rs12925933
rs6881568
rs2977608
rs56799554
rs13062095
rs1023767
rs11412296
rs7099229
rs55747751
rs185174247
rs3891167
rs2230590
rs11557154
rs4695407
rs1985369
rs2555104
rs871134
rs10773176
rs117407747
rs6776756
rs762679
rs12412214
rs10768683
rs1332941
rs1611236
rs10905255
rs17803849
rs4724
rs34550383
rs76065543
rs182059586
rs3785074
rs6536702
rs17677991
rs932002
rs2763979
rs35446936
SNP
−0.050
−0.025
0.000
Effect size (b)
